# Exploring Student Experiences in a Transdisciplinary Clinical Immersion Course on Needs Identification in Veteran and Service Member Healthcare Settings

**DOI:** 10.1007/s43683-025-00201-w

**Published:** 2025-11-13

**Authors:** Sarah Scheerer, Chloe Tenembaum, Aliza M. Lee, Devasmita Choudhury, Brad D. Hendershot, Andre Muelenaer, Ashley R. Taylor, Martha Sullivan, Pamela J. VandeVord, Elham Morshedzadeh, Christopher B. Arena

**Affiliations:** 1 Biomedical Engineering and Mechanics, Virginia Tech, Blacksburg, VA 24060 USA; 2 Salem Veterans Affairs Medical Center, Salem, VA 24153 USA; 3 Virginia Tech Carilion School of Medicine, Roanoke, VA 24016 USA; 4 University of Virginia School of Medicine, Charlottesville, VA 22903 USA; 5 Extremity Trauma and Amputation Center of Excellence, Walter Reed National Military Medical Center, Bethesda, MD 20889 USA; 6 School of Design, Virginia Tech, Blacksburg, VA 24060 USA; 7 Gerald D. Hines College of Architecture and Design, University of Houston, Houston, TX 77204 USA

**Keywords:** Biomedical engineering, Clinical immersion, Biodesign, Needs identification, Healthcare innovation, User-centered design

## Abstract

**Background:**

As the number of BME clinical immersion experiences expands across university curricula, there is a growing opportunity for BME educators to share practical insights gained from implementing clinical immersion courses. Despite the growing scholarship exploring BME clinical immersions, a significant need for robust exploration remains as we work to understand the impact of such programs on student learning.

**Purpose:**

To address this gap, the purpose of this work is to describe the design, implementation, and assessment of a BME course focused on clinical immersions in Service Member and Veteran healthcare environments.

**Methods:**

We designed, implemented, and assessed student experiences in a new technical elective course in our undergraduate BME curriculum entitled *Needs Identification in Healthcare*. This paper analyzes data across the first three cohorts from students’ needs identification experiences, including working in transdisciplinary teams and immersion in Veteran and Service Member healthcare environments. The program structure is described with key elements that include (1) immersion partner collaboration, (2) team-based immersion experiences, (3) needs-finding emphasis, (4) team-based engineering design experiences, and (5) immersion assessment and evaluation. Techniques for student assessment include quantitative and qualitative survey items for investigating the program structure, complementary roles of engineers and designers, needs-finding ability, overall immersion experience, training content, faculty support, team effectiveness, self-reflection, and professional development.

**Results:**

Overall, students had a high appreciation for the clinical immersion experience and benefited from their participation in the course in terms of their ability to problem solve, identify healthcare-related needs of Veterans, communicate with patients and providers, and work effectively in transdisciplinary teams wherein complementary roles of engineers and designers are valued.

**Conclusion:**

Structured clinical immersion experiences that incorporate transdisciplinary teams and scoped healthcare environments promote student learning and professional development.

## Introduction

Clinical immersion is an important aspect of experiential learning in undergraduate Biomedical Engineering (BME) education [1, 2]. Clinical immersion provides students with an opportunity to engage with a variety of stakeholders directly in the healthcare environment, exposing students to the daily challenges of healthcare delivery using a needs-based approach [1, 3]. Direct exposure to the clinical environment enables BME students to foster a deeper understanding of the perspectives of both the patient and the provider. From recent scholarship assessing the impact of clinical immersion programs on BME student learning, cultivation of students’ empathy is emerging as a meaningful element of students’ experience along with user-centered design [3]. For engineers and designers, gaining empathy can facilitate capturing key user needs and constraints in the design process, ultimately resulting in improved medical products and processes [1, 4]. For example, an article by Kotche et al. suggested that clinical immersions support medical device innovation by fostering an environment that can allow students to bring in a new perspective to identify unmet clinical needs [1]. In addition to supporting medical device innovation, clinical immersion opportunities have also demonstrated impact on student learning experiences, including prototyping experiences [5, 6], communication skills [7, 8], and critical-thinking capabilities [7]. Combined, the existing data clearly demonstrate that BME students can benefit from clinical immersion opportunities, with the main driver being an interest in the healthcare field and finding ways to improve it.

Collaborations between BME and Industrial Design (ID) students in clinical settings have shown mutually beneficial relationships that improve project outcomes [9]. In industry, professionals are expected to work across disciplinary boundaries daily to produce creative and functional solutions [10]. By participating in joint courses, students engage in experiences that help them successfully transition into the workforce after graduation. While beneficial, there can be challenges in providing students with these experiences [9]. Strong commitment from faculty, healthcare professionals and administrators is vital for several reasons, such as the time investment, onboarding processes, and coordination of clinical schedules. Benefits to the students are numerous and include learning to communicate in other professional languages, openness to multiple perspectives, and a broader knowledge of design processes [11]. Students are also more likely to continue engaging in interdisciplinary projects after the experience. These skills make students uniquely marketable for industry positions post-graduation as more effective collaborators.

The number of interdisciplinary clinical immersion courses across the country is growing, affording students the opportunity to learn how the different disciplines approach problem-solving and designing solutions [2]. For example, a course at Marquette University brought ID students from the Milwaukee Institute of Art and Design into elements of the BME capstone projects as design consultants [9]. Noted benefits included a mutual respect for complementary skill sets and problem-solving approaches. Select recommendations for successful collaboration included introducing ID topics early in the engineering curriculum, sharing the title of “Designer,” and screening projects for sufficient ID-related work. Students also indicated that working on the project together from the beginning would be advantageous [9]. The University of Cincinnati hosts a cross-disciplinary course focused on biomedical projects [12]. Researchers found that students learned to recognize and appreciate each other’s skills and strengths throughout the 6–9-month experience. Initially, the teams were selected by the instructors and matched to projects without student input. This evolved to matching students based on a team selection questionnaire that focused on skills, career and course goals, and project interest. The instructors learned that students were more motivated and produced better results when they had input on their project placement [11]. Similar interdisciplinary initiatives at Carnegie Mellon University incorporate ID students into BME capstone projects [13]. From this collaborative learning environment, communication dynamics were highlighted as representative of the medical device industry. Additionally, researchers noted that careful thought should be given to the team structure and an equal distribution of workload relative to each discipline [12].

As the number of BME clinical immersion experiences expands across university curricula, there is a growing opportunity for BME educators to share practical learning from implementing clinical immersion programs. Toward this knowledge sharing, recent scholarship has surveyed the landscape of clinical immersion programs across BME curricula to develop a more comprehensive understanding of program format, goals, and learning outcomes [2], ultimately supporting the BME education community in developing clinical immersion opportunities for their own learning contexts. Additionally, recent work from the University of Kentucky and Indiana University–Purdue University Indianapolis has identified five interconnected, essential elements of BME-focused immersion programs, including (1) immersion partner collaboration, (2) team-based immersion experiences, (3) needs-finding emphasis, (4) team-based engineering design experiences, and (5) immersion assessment and evaluation. Students self-reported increased knowledge in identifying and refining user needs, team-based problem-solving, and confidence in concept generation [14]. Despite the growing scholarship exploring BME clinical immersions, a significant need for robust exploration remains as we work to understand the impact of such programs on student learning [2]. To address this gap, the purpose of this work is to describe the design, implementation, and assessment of a BME course focused on clinical immersions in Service Member and Veteran healthcare environments.

### Course Overview

We designed, implemented, and assessed student experiences in a new technical elective course in our undergraduate BME curriculum entitled *Needs Identification in Healthcare*. Launched in Spring 2020, the one-semester course has continued annually except for the Spring 2021 semester due to the COVID-19 pandemic. This paper analyzes data across the first three cohorts from students’ needs identification experiences, including working in transdisciplinary teams and immersion in Veteran and Service Member healthcare environments. Below, we describe the structure and format of the course, learning objectives, and final course deliverables.

#### Course Structure and Format

The *Needs Identification in Healthcare* course was designed as a three credit-hour, 3000 level technical elective course in our undergraduate BME curriculum. The course structure included a combination of faculty and guest lectures, clinical immersions, and student teamwork time to build and analyze a database of needs identified during clinical immersions [15]. The course convened one day per week for 2.5 hours and alternated between time in the classroom and field visits to a regional Veteran Affairs (VA) hospital, with a total of 4–5 clinical immersion days throughout the semester. These immersion days were scheduled according to clinician and specialty availability, and the majority of the visits were after the faculty and guest lectures, which were used as preparation for immersion. The initial lectures provided an overview of the needs-finding process, the specific roles of BME’s and ID’s in industry, and clinical etiquette. Guest lectures and background readings were curated to provide an overview of the disease state fundamentals in three clinical focus areas—nephrology, podiatry, and gerontology. Faculty lectures were developed as a combination of content from the Stanford Biodesign process [16] and contextual inquiry methods [17]. For example, ID faculty focused on user-centered research methods and data analysis and visualization. BME faculty focused on the development of needs statements and needs databases, with additional lectures on regulatory, reimbursement, and business considerations to aid in needs prioritization. The immersion activities varied by specialty. Within nephrology, the students primarily observed dialysis. Within podiatry, the students primarily observed patient care related to foot and ankle surgery, wound prevention, and limb salvage. Within gerontology, the students primarily observed a virtual geriatric fitness program called Tele-Gerofit [18] and had discussions with providers regarding challenges within the field of telemedicine.

At the beginning of the course, the students were presented with background information on each specialty and immersion teams were assigned by interest. These teams included both BME students and ID students in their sophomore, junior, or senior years, who worked together within that specialty for the entire duration of the course. Each specialty had one team for each course offering, except for Spring 2023, which did not have a Tele-Gerofit team, for a total of eight teams across the three years.

Prior to the initial clinical immersion, students completed the appropriate trainings as required by the hospitals. The initial visit to the regional VA hospital consisted of the students finishing their onboarding paperwork, meeting their clinical mentors, and touring their clinical focus area. Subsequent visits consisted of a variety of activities, including technology demonstrations, observing procedures, and interviewing patients. Immersion in a new environment required them to ask clarifying questions, rely on each other’s strengths and backgrounds, and combine their knowledge to create needs statements. Ultimately, these practices helped to uncover potential design opportunities within the VA healthcare system.

An opportunity to visit a large military hospital occurred later in semester and was distinct from the primary needs-finding initiative at the regional VA hospital. While both hospitals participate in research and deliver care to patients, they serve different patient populations and receive funding from different places. The regional VA hospital is funded by the Department of Veteran Affairs and primarily serves military Veterans, while the military hospital is funded by the Department of Defense and primarily serves active Service Members, their families, and military retirees. The visit occurred later in the semester to facilitate student preparation as well as work with visitation restrictions associated with the COVID-19 pandemic. A lecture on military injuries was provided as preparation, along with reading assignments on extremity trauma. During the visit, students met with military health researchers to learn about the latest techniques for optimizing outcomes for Service Members, Veterans, and beneficiaries, such as musculoskeletal conditions secondary to limb loss [19], powered prosthetic devices [20], and osseointegration [21]. The students also toured the associated facilities and observed demonstrations of equipment and research protocols.

#### Course Learning Objectives

Course learning objectives were developed for the initial implementation of the course as shown in Table 1. The learning objectives evolved across the first three cohorts based on feedback from students and course stakeholders. For example, initially the learning objective “prepare a proposal/process book” was included. However, instructors pivoted away from process books after the first year of implementation based on student feedback and focused on a project proposal. The final course learning objectives for the fourth iteration of the course are listed in Table 1, following feedback collected from the first three cohorts analyzed in this study.

**Table 1:** Course learning objectives

Course Learning Objectives, 2020	Course Learning Objectives, 2024
• Create a user-centered research plan • Conduct user-centered research and analysis • Assess the viability of different user needs • Develop problem statements • Prepare a proposal/process book • Demonstrate the ability to carry out an individual or group investigation	• Conduct a needs assessment in a clinical setting using user-centered research methods and consideration of disease state fundamentals • Develop formal problem statements for needs in healthcare • Synthesize needs by considering the technical, market, regulatory, and reimbursement landscapes • Create a proposal to justify further research and development on a high-priority need • Collaborate and communicate effectively on transdisciplinary teams in healthcare

#### Course Deliverables

By the end of the course, students generated a list of unmet needs, formulated official needs statements, and filtered the statements through a prioritization matrix based on practicality and impact. Deliverables included a notebook for documenting insights or observations during clinical immersions or other class lectures or reading assignments, a team contextual inquiry case study [15] review, a team needs database, a team midterm and final presentation, and a project proposal on an individual student’s top identified design opportunity. The midterm presentation allowed students to present their initial findings and receive feedback from the teaching team. Presentation content included an introduction to the clinical focus area and disease state, a description of the clinical environment, a description of the devices utilized in patient care, patient persona profiles, a stakeholder map, a user journey analysis, and an analysis of identified pain points. The final presentation built upon the midterm to produce a full view of the students’ findings, and included additional content on needs statements and needs evaluation and prioritization, as well as potential solution approaches. Presentations were shared with the teaching team as well as to their VA clinical mentors, providing an opportunity for students to present to an interdisciplinary audience.

### Clinical Immersion Context: Service Member and Veteran Healthcare

A distinguishing feature of our BME clinical immersion course is its focus on Service Member and Veteran healthcare through clinical immersion at a regional VA hospital. Students in our course had the opportunity to practice needs-finding techniques at the regional VA hospital by interacting with physicians, patients, nurses, and other healthcare professionals. These hospitals often support large geographical regions, and patients may find it difficult to access these facilities due to long travel distances, health conditions, and family situations. Patients in VA hospitals are primarily military Veterans, with a wide range of healthcare needs. There are a greater percentage of male Veterans compared to female Veterans. As of 2023, female Veterans account for approximately 10% of the Veterans enrolled in the VA healthcare system [22]. Additionally, over 75% of Veterans enrolled in the VA healthcare system are over the age of 45, with almost 50% of the total enrolled Veterans being 65 and older [20]. Service Members and Veterans have circumstances and experiences that make their healthcare needs unique [23, 24]. For example, Service Members and Veteran populations can sustain complex injuries from physical trauma events such as improvised explosive devices (IEDs) and landmines, which have the ability to result in one or more major limb amputations [21]. Additionally, veterans have increased prevalence of post-traumatic stress disorder (PTSD), which leads to a greater risk of comorbidities and health problems such as tobacco use, cardiovascular disease, and metabolic syndrome [21]. Our teams were challenged to recognize innovation opportunities that would benefit patients and/or providers within the military community. Students also visited a large military hospital as an auxiliary learning opportunity in military healthcare research related to improving outcomes after extremity trauma and limb loss.

## Methods

### Institutional Context

The institutional context for this study is an undergraduate BME program at an R1 institution in the southeastern USA. At the time of our writing, the BME program enrolled ~365 students, with ~200 seeking a B.S. in BME and remaining students seeking a minor in BME.

### Participants

This study includes participants from three cohorts of students across three distinct semesters of the* Needs Identification in Healthcare* course. Across the three cohorts, 29 students elected to participate in this study. In total, there were 18 BME students, two other engineering students who received a BME minor and were included in the BME group, and nine ID students. An overview of study participants is provided in Table 2.

**Table 2:** Breakdown of BME and ID student numbers for each cohort

Cohort	Total students	BME students	ID students
Spring 2020	12	7	5
Spring 2022	10	7	3
Spring 2023	7	6	1

### Data Collection

It was determined that this protocol (IRB-20-091) met the criteria for exemption from IRB review under 45 CFR 46.104(d) category 1. To assess student experiences in the course, a mixed-methods survey was developed as a combination of quantitative, Likert-scale questions along with free-response, qualitative questions. The Likert-scale was anchored from from 1—“Strongly Disagree” to 7—“Strongly Agree.” Students completed the course-specific questionnaire using Qualtrics or Question Pro online survey software. Tables 3 and 4 provide an overview of all survey items used to assess students’ experiences in the course. 

Survey items Q3.1 through Q3.15 in Table 3 were collaboratively designed by the two lead instructional faculty who launched the course, drawing from combined experience in biomedical engineering and industrial design learning environments. Given the new program implementation and exploratory nature of the study, questions were designed to broadly explore students’ experiences related to teamwork, academic motivation, and overall learnings. In addition to the instructor-designed survey, student perceptions were assessed using the institutional Student Perceptions of Teaching (SPOT) surveys; these questions are listed in Table 3 as Q3.16 through Q3.28 and in Table 4 as Q11-Q13.

**Table 3:** Likert-scale survey items included in assessment

Q #	Question
Q 3.1	I am passionate about the clinical focus area that I investigated
Q3.2	Industrial designers and biomedical engineers have complementary roles that improve team performance
Q3.3	My role and contributions to the team were valued by my teammates
Q3.4	I felt a sense of ownership in my team's project
Q3.5	I have enhanced my ability to work in transdisciplinary teams
Q3.6	I am better prepared to enter the biomedical workforce and make positive contributions in my respective field
Q3.7	I have deepened my knowledge of the healthcare ecosystem
Q3.8	I have improved my ability to direct a conversation with a range of audiences, including healthcare providers and patients
Q3.9	I am more prepared to verbally explain my research as a result of the final presentation deliverable
Q3.10	I am more prepared to convey research results in written form as a result of the final process book deliverable
Q3.11	The program faculty ensured that we were well-prepared for the clinical immersion experience
Q3.12	Overall, I am pleased that I chose to participate in this course
Q3.13	Our team has identified a need that, if addressed, will benefit many patients and/or healthcare providers
Q3.14	Veterans and active duty military have unique needs compared to the civilian population
Q3.15	The instructional team collaborated well with each other to manage the course and present content
Q3.16	The instructional team was well prepared
Q3.17	The instructional team presented the subject matter clearly
Q3.18	The instructional team provided feedback intended to improve my course performance
Q3.19	The instructional team fostered an atmosphere of mutual respect
Q3.20	Overall, the instructional team's teaching was effective
Q3.21	I have a deeper understanding of the subject matter as a result of this course
Q3.22	My interest in the subject matter was stimulated by the course
Q3.23	The objectives of the course were clearly explained
Q3.24	The textbook or course readings made a valuable contribution to my learning
Q3.25	I improved my ability to problem solve
Q3.26	The out-of-class assignments were educationally valuable
Q3.27	The in-class assignments were educationally valuable
Q3.28	The instructor related theories and concepts to practical issues

A total of 13 free-response questions were included in the survey, as listed in Table 4. Open-ended questions were designed to explore students’ experiences using qualitative data, providing richer insights and opportunities for continuously improving the course.

**Table 4:** Statements and questions in the free-response section

Q #	Question
Q1	Please select your undergraduate major
Q2	Please describe why you enrolled in the program
Q3	Please describe the clinical focus area that you investigated
Q4	If you are a BME student, what did you learn from working alongside ID students? (4.1) If you are an ID student, what do you learn from working alongside BME students? (4.2)
Q5	Please describe what you learned from visiting the clinical facilities
Q6	Please describe elements of the program that you enjoyed
Q7	Please describe elements of the program that you think can be improved
Q8	Please describe the effectiveness of the program in your education
Q9	Please describe what you learned about yourself as a result of participating in this program
Q10	Please describe if this course impacted your future goals
Q11	What did the instructors do that most helped your learning?
Q12	What could you have done to be a better learner?
Q13	Please provide any additional comments regarding the course and/or instructors

In addition to the course-specific survey, students were also requested to complete assessments using the Comprehensive Assessment of Team Member Effectiveness (CATME), an assessment and feedback program developed by Purdue University [25]. One learning objective of the course centered around conducting research investigations within a group setting; therefore, CATME was chosen as an appropriate assessment tool for the team-based elements of the learning environment. Within CATME, students were prompted to provide feedback on the engagement of their fellow team members, as well as reflect on their own contributions to the team. We leveraged CATME’s five validated dimensions of teamwork to assess students’ learning experiences.

### Data Analysis

A combination of quantitative and qualitative analyses were leveraged for assessment of the course. For survey items using an anchored scale, descriptive statistics were analyzed, including the mean and standard deviation. Additionally, two sample *t* tests were conducted to explore statistically significant differences between BME and ID student responses, with an assumption of homogeneity of variance across the samples (*p*<0.05). SPOT survey questions were not included in the analysis for this study, with the exception of Q3.19 and Q3.25, which were included in the analysis due to the close alignment between the survey item and the specific context of the course. CATME data were categorized into CATME’s five validated dimensions of teamwork, and a composite score was assigned to each category. These categories included “Contributing to the Team’s Work,” “Interacting with Teammates,” “Keeping the Team on Track,” “Expecting Quality,” and “Having Relevant KSAs (Knowledge, Skills, and Abilities).” To display the distribution of data, the average scores from both BME and ID students' perspectives were compared using a box and whisker plot, which allows for a visual examination of the data spread and variations between the two groups.

**Fig. 1:**
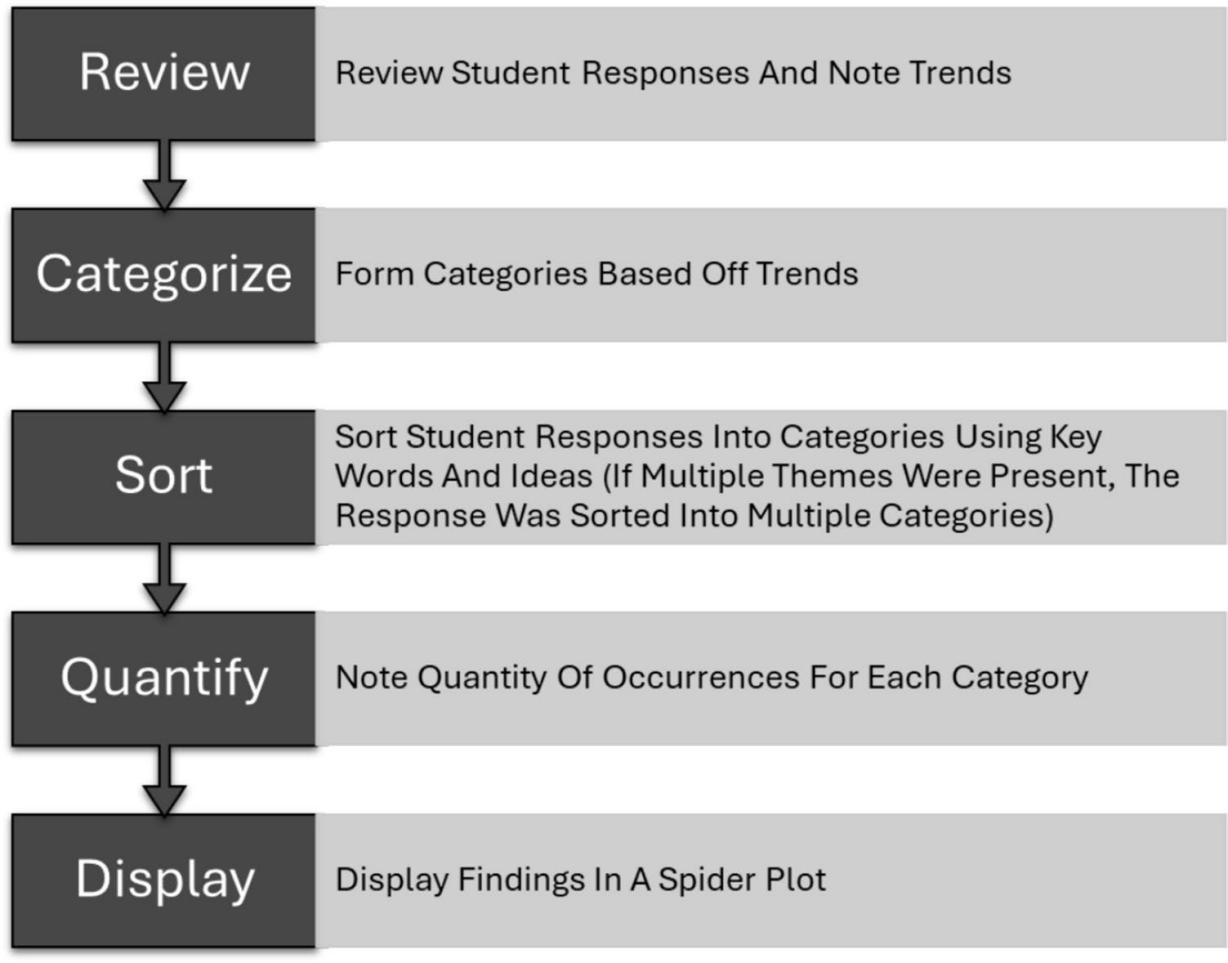
Visual representation of thematic analysis used to analyze qualitative data

From the 13 free-response questions, seven were chosen for further examination (Question 4 was broken up into Questions 4.1 and 4.2). These questions were chosen based on the rich and thorough nature of student responses, which afforded a robust qualitative analysis process. Qualitative data was coded inductively using an expanded thematic analysis process [26]. This process is displayed in Fig. 1 for clarity. Responses and their assigned categories were reflected in radar plots.

## Results

Below, we provide an overview of results from assessing the BME clinical immersion course across three cohorts. We first discuss results from quantitative analysis of anchored scale questions, highlighting statistically significant differences between BME and ID students where applicable. Following, we discuss themes from the qualitative, open-ended survey items. We then provide an overview of CATME results comparing BME and ID students across dimensions of teamwork. Finally, we conclude our results section with a summary of clinical needs identified through the course and examples of student-generated course deliverables.

### Quantitative Results from Anchored-Scale Survey Items

**Fig. 2:**
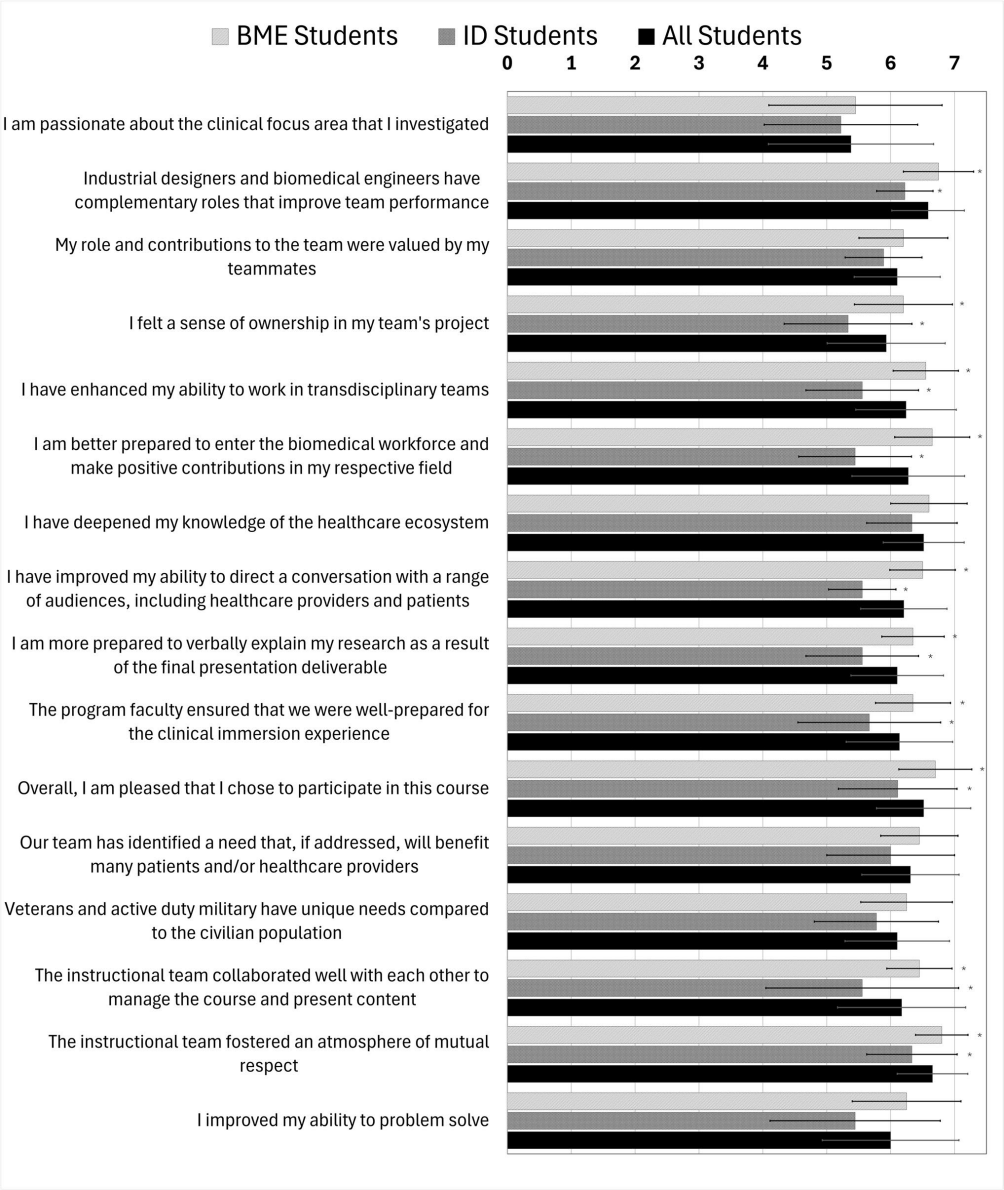
Likert multiple-choice data including scores from BME students, ID students, and all students combined. The error bars represent +/− 1 standard deviation

To compare the Likert-to-numerical scale data derived from the statements in the course-specific survey, a bar graph was generated (Fig. 2). The scores obtained for each question were consistently high, resulting in an overall average of 6.2. It was found that the statements “The instructional team fostered an atmosphere of mutual respect” and “IDs and BMEs have complementary roles that improve team performance” received the highest overall scores and exhibited the lowest standard deviation. Conversely, the statement “I am passionate about the clinical focus area that I investigate” obtained the lowest overall score, while also displaying the highest standard deviation. Across all questions, BME students showed greater agreement with the statements compared to ID students. Statistically significant differences between BME and ID scores for questions are indicated by an asterisk (*) to the right of the bar graphs.

### Qualitative Results from Open-Ended Survey Items

**Fig. 3:**
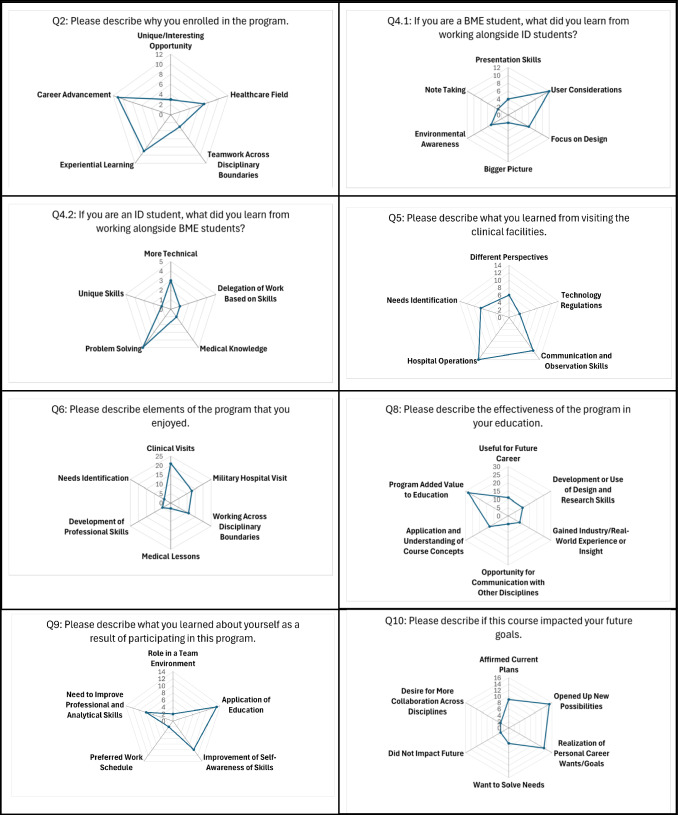
Spider plots from student free-response data, documenting key answer themes

Free-response Questions 2, 4, 5, 6, 8, 9, and 10 were selected for analysis and graphing in spider plots (Fig. 3). In Question 2, the responses were categorized based on different motivations for enrolling in the program. The category with the highest number of responses, totaling 11, was “Career Advancement.” Following closely behind was “Experiential Learning,” with nine responses. On the other hand, the categories of “Unique/Interesting Opportunity” and “Teamwork Across Disciplinary Boundaries” were not mentioned as often, with three responses each. For Question 4, which focused on what BME and other engineering disciplines learned from working with ID majors, the most frequently mentioned factor was “User Considerations,” with 12 responses. The categories of “Note Taking” and “Bigger Picture” were less common in BME student responses. Regarding ID majors’ perspective on working with BME and other engineering disciplines, the most reported learning aspect was the emphasis on problem solving, with five responses. Additionally, three responses highlighted the importance of a more technical focus that biomedical engineers bring. In Question 5, which explored what respondents learned from the clinical visits, the majority of participants reported gaining insights into “Hospital Operations,” with 14 responses. The second most mentioned category was “Communication and Observation Skills,” with 11 responses. For Question 6, when asked about their most enjoyed aspect of the course, 21 students expressed their appreciation for the clinical visits, while 13 students mentioned their enjoyment of the military hospital trip. Question 8 showed that almost all students found that the program added value to their education, with 28 students including this sentiment in their answers. Additionally, 13 students chose to emphasize that the program was effective in the application and understanding of the course concepts. A total of 11 students mentioned that their experience was useful for their future career. Many students also appreciated that they were able to develop and use their design and research skills, with this appearing in 10 responses. On a similar topic to future careers, eight students noted that they were able to gain real-world experience or insight as a part of this program. Finally, five students highlighted that they appreciated the opportunity to communicate and collaborate with other disciplines.

In Question 9, the majority of students reported learning how to apply their education, with 13 responses. Additionally, eight students acknowledged the need to improve their professional and analytical skills. Lastly, Question 10 asked students about their future goals and how the course made an impact on them. Out of the total responses, 23 students felt that the course had impacted their future goals, three were unsure, and three stated that there had been no impact. For the common themes analyzed in the radar plots, 15 students felt as though the course opened them up or steered them to new possibilities, while nine students felt that their current plans had been affirmed. Ultimately, 13 students mentioned that they realized personal career wants/goals from their experience. In their answers, five students listed that they want to solve needs in future and three stated that they want to do more work collaborating across disciplines. Overall, by analyzing and graphing the selected free-response questions from the course-specific survey, valuable insights were obtained regarding students’ motivations, learning experiences, and preferences throughout the course.

Free-response Question 2 asked students to describe their reasons for enrolling in the course. As seen in Table 5, common themes based off key words and ideas presented by the students included *Experiential Learning, Career Advancement, Healthcare Field, Teamwork Across Disciplinary Boundaries, and Unique/Interesting Opportunity*

**Table 5:** Themes in students' responses to Question 2

Question 2: Please describe why you enrolled in the program	
Theme (Occurrences)	Example Key Words and Ideas
Experiential Learning (9)	Hands-On
	Real-World
	Experience
	Learning
Career Advancement (11)	Job
	Career
	Medical Field Aspirations
Healthcare Field (7)	Medical Field (Without Mentioning a Career in Medicine)
	Clinical
	Health
Teamwork Across Disciplinary Boundaries (3)	Interdisciplinary
	Different Majors
Unique/Interesting Opportunity (3)	Cool
	Unique

Free-response Question 4 prompted students to reflect on learnings from their interdisciplinary peers (i.e., 4.1: If you are a BME student, what did you learn from working alongside ID students? 4.2: If you are an ID student, what do you learn from working alongside BME students?) Each set of responses were analyzed separately. Table 6 shows the key words and categorization process for Question 4.1. These categories were *Presentation Skills, User Considerations, Focus on Design, Bigger Picture, Environmental Awareness, and Note Taking*.

**Table 6:** Themes in students' responses to Question 4.1

Question 4.1: If you are a BME student, what did you learn from working alongside ID students?	
Theme (Occurrences)	Example Key Words and Ideas
Presentation Skills (4)	Aesthetic/Effective Presentation
	Preparing Information for Audiences
	Communication to Audiences
	Presentation
User Considerations (12)	Stakeholder
	User
	Human Factors
	Emotions
Focus on Design (6)	Design
Bigger Picture (2)	Comprehensive Approach
	Bigger Picture
Environmental Awareness (5)	Environment
	Space
	Surroundings
Note Taking (3)	Visualizing Information
	Mapping
	Taking Notes
	Sketches
	Drawing

Table 7 shows the key words for Question 4.2’s categories, which pertain to answers from ID students. These categories were *More Technical, Problem Solving, Medical Knowledge, Delegation of Work Based on Skills, and Unique Skills*.

**Table 7:** Themes in students' responses to Question 4.2

Question 4.2: If you are an ID student, what did you learn from working with BME students?	
Theme (Occurrences)	Example Key Words and Ideas
More Technical (3)	Technical Side
	Technical Solutions
	Complex and Technical Solutions
	Learning
Problem Solving (5)	Problem Solving/Analysis
	Approach to Problems
	Quick to New Ideas
Medical Knowledge (1)	Knowledge of Topics
Delegation of Work Based on Skills (1)	Divide Work with Different Skills
Unique Skills (1)	Skills Unique to Discipline

Regarding free-response Question 5, which asked students to describe what they learned from visiting the clinical facilities, responses were grouped into five categories: *Different Perspectives, Hospital Operations, Needs Identification, Communication and Observation Skills, and Technology Regulations*. Table 8 displays these categories and the key words and ideas from student responses that would place an answer in that category.

**Table 8:** Themes in students' responses to Question 5

Question 5: Please describe what you learned from visiting the clinical facilities	
Theme (Occurrences)	Example Key Words and Ideas
Different Perspectives (6)	Communication with Doctor
	Patient Care
	Learned About Problems Experienced by Others
	Emotions
	Communication Between Professionals
Hospital Operations (14)	Hospital Structure
	Task Assignments Between Professionals
	Stakeholders
	Environment
	Process/Steps
	Facility
	Understanding of Operations
Needs Identification (8)	Needs
	Problems
	Identify
	Solution Generation
Communication and Observation Skills (11)	Observing
	Asking Questions
	Interviewing Skills
	Watch
	Data Collection Methodology
	Etiquette And Professionalism
	Communicating
Technology Regulations (3)	Regulations
	Security
	Design Constraints
	Process of Developing/Implementing Devices

To evaluate the program’s highlights, free-response Question 6 asked students to describe elements of the program they enjoyed. Based on the student responses, as seen in Table 9, the categories were determined to be,* Clinical Visits, Military Hospital* *Visit,* *Working Across Disciplinary Boundaries, Medical Lessons,* *Development of Professional Skills, and Needs Identification*. If students mentioned specific locations such as the “VA [Veteran Affairs Hospital],” “Medical Setting,” “Clinical Setting,” “Hospital,” the mentors at the VA hospital, or interactions with clinical staff, their responses were categorized as “Clinical Visits.” Since the students visited two hospitals, the regional VA Hospital and the military hospital, we needed to differentiate between them. To do so, we assumed that if only one clinical facility or hospital was mentioned, it referred to the VA Hospital since we visited there more frequently. If the responses mentioned clinical facilities in the plural form, it was assumed that they were referring to both the VA and military hospitals. Consequently, these answers were categorized under both the “Clinical Visits” and “Military Hospital Visit” categories.

**Table 9:** Themes in students' responses to Question 6

Question 6: Please describe elements of the program that you enjoyed	
Theme (Occurrences)	Example Key Words and Ideas
Clinical Visits (21)	VA [Veteran Affairs Hospital]
	[Military Hospital]
	Medical Setting
	Clinical Setting
	Hospital
	Interactions with Clinical Mentors/Staff
	Facilities
	Visits/Experiences
Military Hospital Visit (13)	[Military Hospital]
	Facilities
	Tour
Working Across Disciplinary Boundaries (11)	Other Students [BME or ID]
	Perspectives
	Collaborative Team
	Working with Another Discipline
	Learning [About BME or ID]
	Transdisciplinary
	Team
Medical Lessons (3)	Medical
	Content
	Learning
Development of Professional Skills (5)	Practice
	Companies
	Better Engineer
	Research
	Improvement of Problem Solving
	Careers
	Information Gathering
	Data Analysis
Needs Identification (4)	Needs
	Identify
	Troubleshooting

Question 8 asked the students to describe the effectiveness of the program in their education. This was a less structured question, designed to allow students to emphasize the ways in which the course had impacted them. Results are highlighted in Table 10. Students focused on various aspects of the program, with the leading concepts being, *Useful for Future Career, Development or Use of Design and Research Skills, Gained Industry/Real-World Experience or Insight, Opportunity for Communication with Other Disciplines, Application and Understanding of Course Concepts, and Program Added Value to Education*. The category, “Not Effective,” was not included, as no students mentioned that the program was not effective.

**Table 10:** Themes in students' responses to Question 8

Question 8: Please describe the effectiveness of the program in your education	
Theme (Occurrences)	Example Key Words and Ideas
Useful for Future Career (11)	Career
	Future
	Possibilities for Major
	Knowledge for Job
	Reference to Future Careers
	Industry
	Job
	Biomedical Engineers
	Direction
	Insight
Development or Use of Design and Research Skills (10)	Design Research
	Interviews
	Data
	Design Process
	Analysis
	Techniques
	Observation
	Research
Gained Industry/Real-World Experience or Insight (8)	Industry
	Real-Life
	Experiential Learning
	Clinical Setting
	Real
	See Technology
	In Practice
	Observing
	Comparison Between Course and Career
Opportunity for Communication with Other Disciplines (5)	Team Dynamic
	Communicate
	Different Groups
	Others
	Disciplines
	Different Perspectives
Application and Understanding of Course Concepts (13)	Design Research
	User Journey
	Concepts
	Reference to Need Identification
	Reference to Regulatory Bodies or Requirements
	Device Design
	Understanding
Program Added Value to Education (28)	Useful Information
	Learning
	Practice
	Education
	Insight
	Effective
	Important
	Prepared
	Confidence
	Knowledge/Understanding
	Experience
	Taught
	Value
	Develop

Free-response Question 9 asked students to describe what they learned about themselves as a result of participating in the program. The students’ answers could be grouped in the following categories: *Need to Improve Professional and Analytical Skills, Role in a Team Environment, Application of Education, Improvement of Self-Awareness of Skills, and Preferred Work Schedule*. The key words and ideas for each category can be seen in Table 11.

**Table 11:** Themes in students’ responses to Question 9

Question 9: Please describe what you learned about yourself as a result of participating in this program	
Theme (Occurrences)	Key Words and Ideas
Need to Improve Professional and Analytical Skills (8)	Improve
	Learn
	Practice
	Expression of a Lack of Confidence
	Work on in the Future
Role in a Team Environment (2)	Working in a Team
	Role [in the Context of a Team]
	Sharing Opinions
Application of Education (13)	Education
	Solve
	Applicable to Field
	Biomedical
	Design
	Studied
	Learned
	Role [in the Context of a Career]
	Working [in the Future]
Improvement of Self-Awareness of Skills (10)	Greater Knowledge Than Previously Thought
	Confidence
	Capable
	Strengths and Weaknesses
	Handle
	More to Learn
Preferred Work Schedule (2)	Morning Person

Question 10 evaluated whether the students felt as though the course had an impact on their goals for the future. Most students responded with a yes, maybe, or no, and almost all elaborated on their thoughts. The students’ thoughts can be separated into six main categories in Table 12, which include *Affirmed Current Plans, Opened Up New Possibilities, Realization of Personal Career Wants/Goals, Desire for More Collaboration Across Disciplines, Did Not Impact Future, and Want to Solve Needs*.

**Table 12:** Themes in students’ responses to Question 10

Question 10: Please describe if this course impacted your future goals	
Theme (Occurrences)	Example Key Words and Ideas
Affirmed Current Plans (9)	Confirmed
	Same Goals
	Confidence in Current Plans
	Reinforced
	More Interested
Opened Up New Possibilities (15)	Searching
	New Opportunity Found by Participation in Course
	New Interest
	Furthered Interest
	New Career Paths
	New Path
	New Direction
Realization of Personal Career Wants/Goals (13)	Love to Work in…
	Searching for Position
	Confirmed
	Want to Pursue
	Desire
	See Myself
	Furthered Interest
	Firmed
	Improved Understanding
	Direction
	Passion
Desire for More Collaboration Across Disciplines (3)	Collaboration
	Engineering and ID
Did Not Impact Future (3)	Don’t Think Future Goals Were Impacted
	Not Right Now
Want to Solve Needs (5)	Need
	Meet Needs
	Improve Healthcare
	Improve Lives
	Positive Impact

### Teamwork Results from CATME Data

**Fig. 4:**
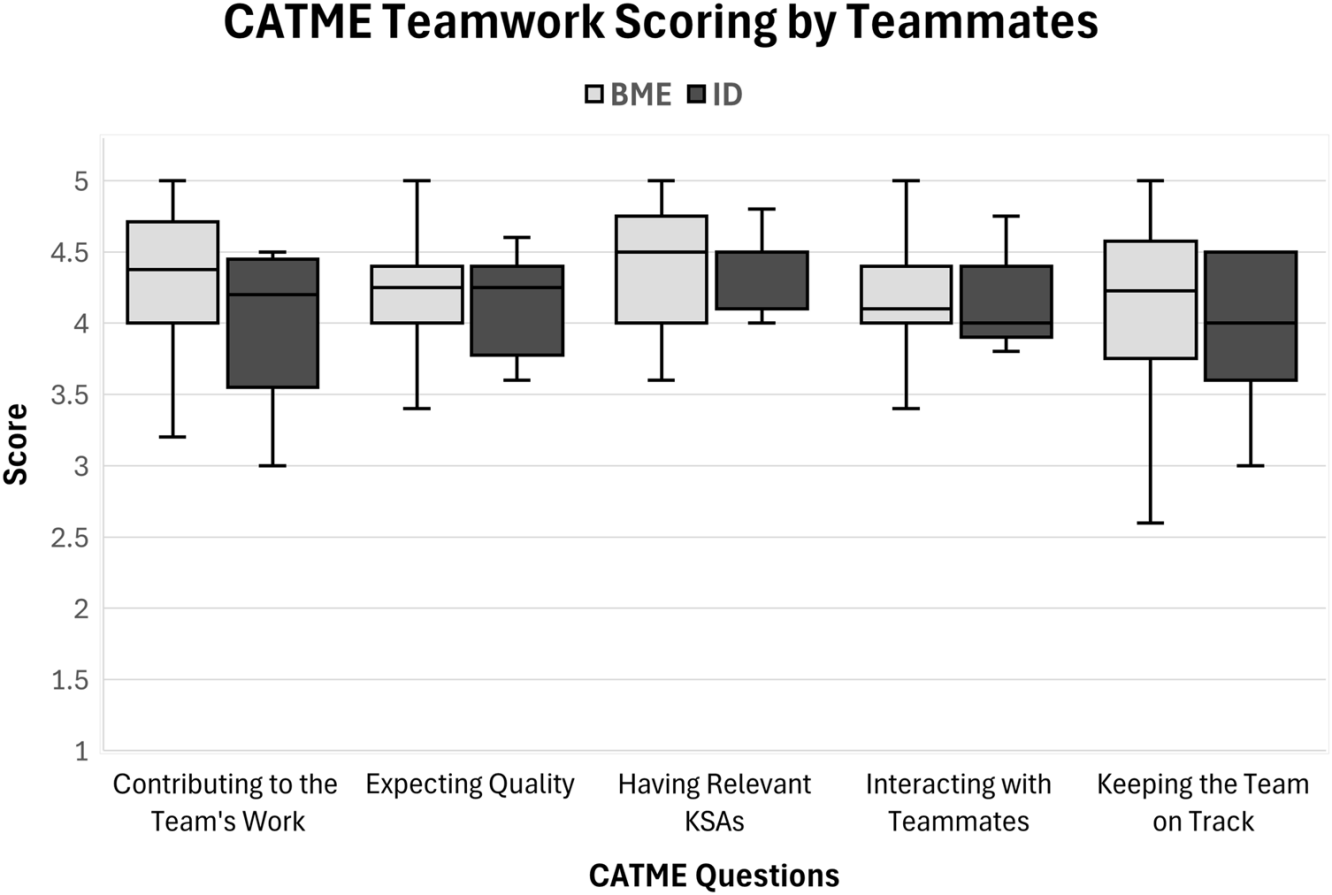
CATME scores from teammate assessment

The CATME Scores encompassed five categories of questions, and their composite scores were visually represented in a box and whisker plot (Fig. 4). The overall mean score across all categories and majors was calculated to be 4.2. On average, as scored by their teammates, BME majors received slightly higher scores compared to ID majors. BME majors had an overall mean score of 4.2, while ID majors had an overall mean score of 4.1. It is worth noting that the sample size for BME majors was larger, but they also exhibited a higher level of deviation in their scores.

### Clinical Needs Identified in Course and Example Student Course Deliverables

**Fig. 5:**
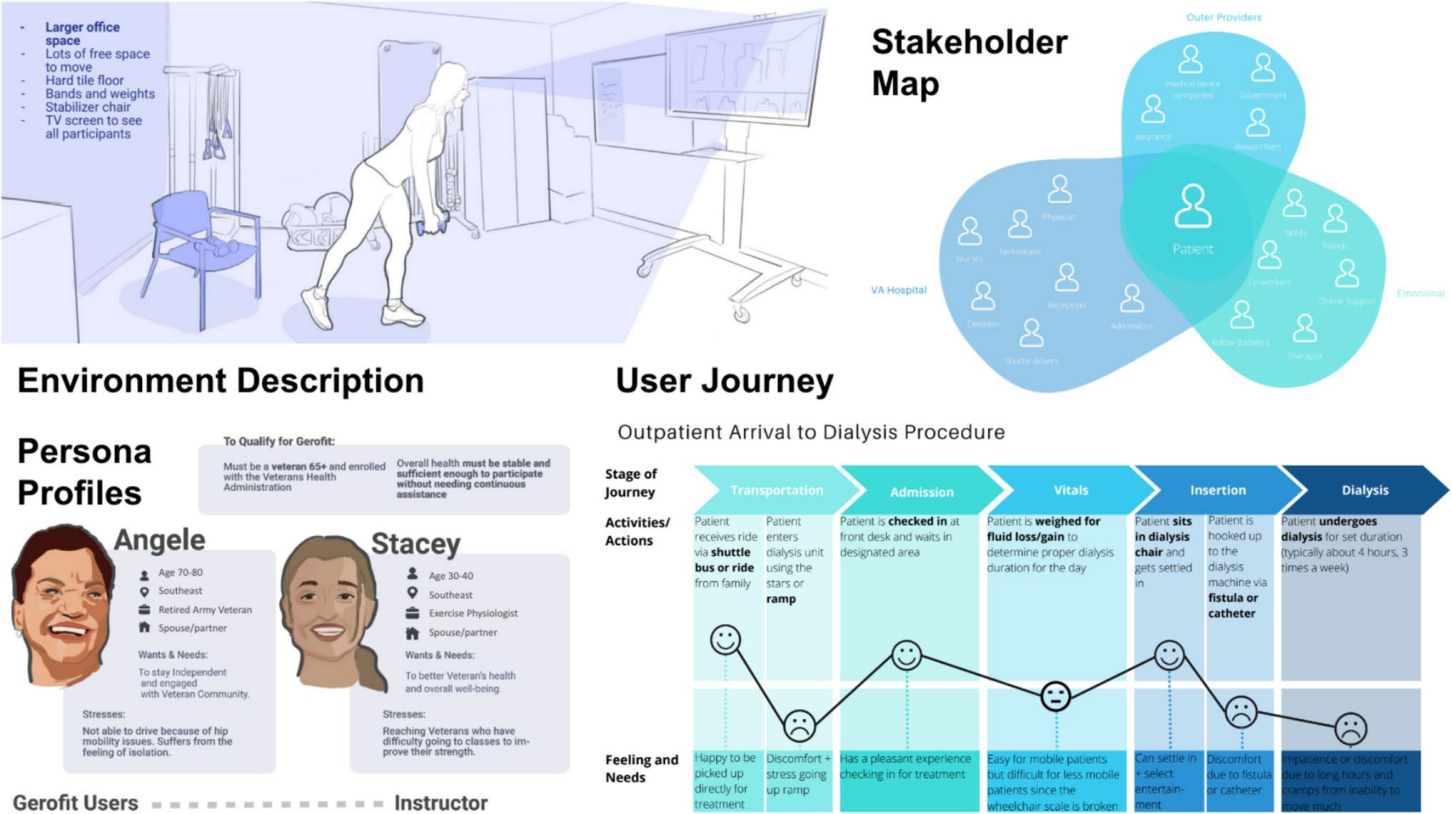
Examples of student work from the Tele-Gerofit (left) and Nephrology (right) teams. The authors affirm that students provided informed consent for publication of the images

Throughout the 2020, 2022, and 2023 years of this course, a total of 206 needs statements were identified with 64 needs in 2020, 63 needs in 2022, and 79 needs in 2023. Examples of student work are shown in Fig. 5 for the Tele-Gerofit and Nephrology teams and example needs statements from all clinical focus areas are shown in Table 13. From the Spring 2020 cohort, one needs statement resulted in an independent research project in collaboration with course participants, instructors, and a VA mentor. Two needs statements were selected as BME senior design projects in a later year (2021–2022). Both included student leaders that participated in the course and VA mentors in different capacities, one as a primary advisor and another as a subject matter expert. Additionally, in one case, the needs statement was broadened by the team to address multiple outcomes. From the Spring 2022 semester, one needs statement resulted in a BME capstone project that was led by a participant from the course with their VA mentor as the primary advisor. From the Spring 2023 semester, one needs statement resulted in a BME capstone project, again with a student lead from the course and continued advisement from their VA mentor. Continued engagement with the VA mentor through the follow-on design phase generated excitement and reinforced the collaboration with the VA during needs assessment activities in subsequent years. BME capstone projects are made up of 4–5 team members, one of which is selected as the team lead. The selection of capstone projects each year by rising seniors is a survey process wherein students rank their top project choices from a competitive pool of faculty and industry sponsored projects. Select, high-priority needs statements with mentor support were selected for the project pool, and based on student enrollment and other project sources, there was not an immediate need to carry additional needs statements from the course forward into capstone.

**Table 13:** Representative needs statements in each clinical area (not necessarily linked to follow-on projects)

Clinical area	Representative needs statements
Podiatry	• A way to normalize pressure distribution in diabetic foot ulcer patients to heal and prevent ulcer formation • A way for patients with neuropathy to know when they are exposed to extreme temperatures to reduce the risk of burns and thermal injuries
Nephrology	• A way to decrease the stress of hemodialysis to improve patients’ emotional well-being • A way to support the connection of tubing for peritoneal dialysis to decrease risk of infection and to increase independence of patients with other disabilities
Gerontology	• A way for at home Tele-Gerofit users to maintain the social benefits of group exercise while operating on a telehealth interface • A way to reduce the amount of the trainer’s technology-handling during telehealth classes to promote safe and efficacious instruction

## Discussion

Designing, implementing, and assessing student experiences in our *Needs Identification in Healthcare* course provided insights into five key areas for clinical immersion courses in BME education: (1) opportunities for assessing learning in clinical immersion experiences, (2) the value of interdisciplinary collaboration, (3) career bridging opportunities for industry, (4) transformative clinical learning experiences within a Veteran and Service Member context, and (5) student perspectives of course effectiveness. Below, we discuss each of these areas, highlighting connections to existing literature and opportunities for future work.

### Assessing Clinical Immersion Courses

Assessing the impact of clinical immersion courses on student learning remains a challenge in BME education [2]. In our experience, leveraging a mixed-methods approach for course assessment data collection provided insights into the benefits and opportunities for improvement of this course. This information will be useful for those considering future clinical immersions in undergraduate courses and/or collaborations between BME and ID, particularly given the similar programmatic elements that many clinical immersion courses exhibit across curricular contexts. For example, our program structure and assessment tools exhibit many similarities to the University of Kentucky and Indiana University–Purdue University Indianapolis run immersion programs [13]. Programmatically, there is (1) a hospital-based immersion with the opportunity for student-patient interaction, (2) a team of two or more students to provide accountability and reassurance [27, 28], (3) a core emphasis on needs-finding and supporting instruction, (4) engineering design instruction and iterative solution development (e.g., follow-on capstone course), and (5) the use of survey instruments for program feedback and student self-assessment. Specific to the assessment tools, programmatic questions investigate structure, contents, delivery, instruction, experience with providers (i.e., mentorship), and overall immersion experience. Combined, our experience implementing and assessing this course along with existing literature highlights the importance of assessing the immersion experience, training content, and faculty support.

Overall, our assessment data suggest that the clinical immersion course benefited students’ learning in terms of their ability to problem solve, identify needs (including those unique to Veterans), communicate with patients and providers, and work effectively in transdisciplinary teams wherein complementary roles are appreciated. Similar assessment mechanisms in [13] resulted in similar themes, such as students self-reporting increased knowledge in identifying and refining user needs, team-based problem-solving ability, and confidence in concept generation. Our assessment data pointed to multiple elements of academic motivation (e.g., [29]) that were cultivated in the course experience. Students’ articulated high appreciation for the value of the clinical immersion experience (our most common response in Question 6, Fig. 3). For example, a student noted, “This was my favorite part of the course. Each visit I learned something new.” Moreover, many students showed that they had developed a passion for solving problems in healthcare. A student noted that, “The knowledge we gained from these visits was used to develop numerous pressing needs that should be addressed to improve dialysis and stakeholders well-being.” The hardships that they observed created an ambition to have a positive impact on quality of life. Another student commented, “although it was a scary experience sometimes, and it was emotionally difficult I think it would be very rewarding to design devices to help people in these situations such as needing to use dialysis to treat kidney failure.” The self-discovery that these students experienced during their semester of needs finding shows promise of a lasting impact on their future. Summing up their experience and combining passion and empathy, a third student stated, “I really enjoy medicine and I hope I can make devices that affect people for the better.” These students completed the course with a greater understanding of the challenges being experienced and turned that understanding into motivation to make a difference in the lives of those around them. Lastly, a student shared, “I felt like I was actually doing something real that can help real people. It was exciting and brought me a new sense of purpose.” Clinical immersion provides an opportunity for students to see and be motivated by the real-world lasting impact that they can have through health innovation. Altogether, this data ultimately suggests that clinical immersion courses may create a learning environment that is academically motivating, leveraging multiple principles of academic motivation theory, including empowerment, usefulness, success, interest, and care from instructors [27].

### Value of Interdisciplinary Collaboration

A major focus of our course was cultivating interdisciplinary collaboration between BME and ID students. The overall high teamwork skill scoring indicates that the students learned to act collaboratively as a team across different majors. The CATME scores, where the students rate themselves and other team members, show that ID and BME students ranked each other similarly (Fig. 4). Overall, ID students had slightly lower scores, but these teamwork metrics may be more targeted toward engineering teamwork metrics. Further research is warranted to compare he perceived value of clinical immersion courses from the perspective of ID and BME to determine whether ID students value different aspects of a project compared to BME students. Additionally, the slightly lower teamwork scores for ID students may have been exacerbated, as there were more BME students in the class and therefore not an equal number of BME students rating ID students compared to ID students rating BME students. Encouraging more ID students to join the course in its recruitment process could close the gap between BME and ID student numbers and satisfaction. Ultimately, all students were satisfied with their teammates regardless of major, with the average of all questions being above a 3.9 out of 5.

Qualitative data echoed the CATME survey results. A student stated in response to Question 6, “I enjoyed working in a team the entire semester and getting to know one another and learn about each of our unique interests and skills.” Students recognized the value that the different education focuses brought to the table. This is consistent with the benefits found in other transdisciplinary courses [4, 5]. Relative to elements of the program that students enjoyed, an ID student stated, “I know that was the whole purpose of this class, but I just found it really helpful and intriguing to learn how we all approach problems and how ultimately we can do things alone, but the best outcomes are from this collaboration and challenging each other.” A BME student echoed this sentiment in their response, writing “As an engineering student, I also greatly enjoyed learning about ID and I now feel that I am a better engineer and problem-solver because of it.” Many BME students stated that they learned about the need to consider the user experience and incorporate human-centered design into their work (Question 4.1 in Fig. 3). This indicates the importance of the lectures given by Industrial Design faculty throughout the semester on these topics and the ability to practice the skills during clinical immersions as well as on assignments. Furthermore, students in BME majors may benefit from the addition of these topics into their standard coursework. At the end of the course, students were able to, as a whole, identify that BME students were described mainly as problem solvers whereas ID students were described as concentrated on the user and user experience. To fully realize the potential of a transdisciplinary course, instructors should emphasize interaction between students, valuing interdisciplinary expertise, and strength identification within teams. For example, fostering both planned, in-class discussions and impromptu discussions on complementary skillsets and academic training and resources facilitated a deeper understanding of the roles that BME and ID students play in the needs assessment process.

### Clinical Immersion Experiences as Career Bridging

The program encouraged career growth in the majority of participants as well as informed future career plans. The students felt encouraged to pursue opportunities working in transdisciplinary teams after the conclusion of the course, with a student stating in free-response Question 10, “This course has made me want to pursue a job that involves contextual inquiry and collaborations with ID.” Relating to career plans, many students shared that this course was beneficial to making decisions about their future. Some students felt that the course affirmed their current career goals, while others found that the course directed them to consider different career possibilities. One student shared, “This course impacted my future goals because it gave me a direction that I would like to go with BME. It enhanced my interest in biomechanics and devices and sparked my interest in the possibility of me wanting to go to grad school.” Another student wrote, “I feel it reinforced and grew my passion for improving medicine and health.” Students discovering that they do not enjoy something can be a positive revelation. Two students noted this in their responses, with one saying, “I have realized that I am more interested and probably better suited for medical device design and development as opposed to research,” while the other commented that the course, “firmed up my belief that I do not want to be a medical doctor.” These student’s experiences further emphasize the career benefits that immersion provides students, and they provide encouragement for universities considering incorporating clinical immersion into their programs. In our assessment plans for future iterations of the course, we plan to include longitudinal follow-up data collection with students to understand their career outcomes and how the program may have impacted their plans.

### Transformative Learning in Veteran and Service Member Healthcare Environments

One theme that emerged in student feedback was the value of engaging in clinical immersions in the VA clinical environment. Though﻿ BME clinical immersion programs are expanding across the country, at the time of writing, there is a dearth of literature on BME clinical immersion courses in this context. For our course, the partnership with the VA emerged from faculty collaborators, who shared a passion for supporting this patient population and have research expertise in areas that often intersect with the unique needs of Veterans (for example, in traumatic brain injury). Currently, we are not aware of any other BME clinical immersion program that partners with Veteran healthcare environments. However, existing curricular innovation in the nursing environment suggests that mobilizing learners to deeply understand the needs of Veterans and Service Members may be a ripe opportunity for expanding student learning experiences in biomedical engineering education. For example, Flores et al. developed a simulation learning experience for nursing students to explore a case study on obstetrics healthcare needs for a 23-year-old Iraq combat Veteran [30]. From this simulation exercise, nursing learners indicated a newfound empathy for the needs of Veterans as well as an enhanced understanding of the complexities of Veteran care [28]. This data is striking given that Veterans represent a medically underserved population, particularly in rural areas [32], with studies suggesting that Veterans have significantly higher rates of heart disease, stroke, cancer, COPD, and obesity as compared with non-Veteran status [30]. Given these growing health disparities, developing community-based partnerships with Veteran and Service Members’ healthcare environments may be a powerful opportunity for BME education to more actively contribute toward health equity. Ultimately, exposing students to the healthcare needs of underserved populations, such as Veterans and Service Members, heeds the recent warning from Lanier et al. [33]: “*as biomedical engineers developing the next generation of healthcare technologies, we are poised to either improve the health disparity landscape or further widen the gap.*”

Although not specifically explored in the questions that were asked, the students’ answers to the question set demonstrated that this course fostered empathy within the students by seeing and hearing first-hand what challenges are faced in VA healthcare environments. One student emphasized this point by saying, “Visiting clinical facilities helped to give a better sense of who the stakeholders are, what real problems they face, and what current solutions are available. Getting out of the classroom drives home the point that these are real people with real problems.” Interactions with patients and providers and personally observing hardships can help students connect with their work and motivate them to deliver better solutions. A student noted that they left the course with a “much better understanding of the emotional side of caring for patients, this is an important factor that many engineers ignore during the design process.” This sentiment was also shared with ID students, with one of them stating, “I think seeing the environments and speaking to the doctors about these topics really helped us both visually and auditorily understand the needs that are required in this industry.” Another student commented that, “As a future biomedical engineer, the patient has to constantly be in mind.” Because our study was not designed to compare different types of clinical environments (e.g., VA vs. other types of clinical environments), we are not able to draw comparative claims from our data. Our future implementations of the course will include qualitative assessment strategies that enable comparison of student experiences across clinical immersion environments to fully illuminate elements of clinical immersion that may be unique to the VA environment.

#### Student Perspectives of Course Effectiveness

Overall, our assessment data suggests course effectiveness in supporting student learning. The variety of student answers shows that students valued different experiences within the course. As previously stated, Question 8 was designed to be open-ended to learn more about what the students felt their greatest takeaways were. In total, 28 responses shared that the course added value to their education in some way, with students commenting that it added, “immense value” and was, “incredibly helpful in my education”. Many students shared that they would be taking the knowledge from the course into their future careers. One student stated that they had experiences that they would, “take into my real job in the coming months,” showing that clinical immersion and interdisciplinary teamwork helps students feel set up for success. Another student commented that the class was, “extremely helpful both in preparation for senior design and for in industry.” From these quotes, students expected long-term benefits following their experience. An additional strong takeaway for students was the application of course content. According to one student, they “learned more about how to approach needs identification and gather research for device design.” Another student stated that they were “glad to get more experience observing and analyzing issues and learning how to find unspoken issues.” This shows that the course lectures combined with the hands-on application of course material was effective. Additionally, the visit to the military hospital was the second highest element of the program that students enjoyed, after clinical visits (Question 6 in Fig. 3). It is important to note that even having one opportunity to visit this military hospital had a large enough impact on the students' experience that they noted it in this question. Lastly, communication was a topic that came up in the survey responses. One student stated, “I think hearing about everyone’s role and how industry teams work is one thing, but to really experience it brings a whole new level of understanding and respect for the team dynamic.” A different student conveyed a similar sentiment saying that the class, “truly prepared me for a key aspect of my future career (the ability to communicate with other disciplines).” The theme of communication across disciplines and within transdisciplinary teams was commonly brought up in student’s answers and shows a positive impact of this class.

## Limitations and Future Work

 Our assessment data collection did not enable comparison of different types of clinical environments (e.g., VA vs. other types of clinical environments). Given there were only VA environments in our study, we are not able to make claims about how VA environments are unique from other types of clinical environments. In the future, our course assessment will include qualitative assessment strategies that enable comparison of student experiences across clinical immersion environments.

Secondly, the ratio between BME and ID students varied in each of the three cohorts. The majority of the students each year were biomedical engineers. This may have been due to the ID student schedule, as sophomore ID students had a required course that overlapped with the time of this course. Additionally, there was a competing elective offered at the same time for ID students of other years. Furthermore, a greater number of ID students opted out of having their data used. These two combined factors resulted in a lower number of data points for the ID students for both the course-specific and CATME scores. Additionally, while the first two cohorts had three clinical focuses, there were only two clinical focuses for the third cohort due to the smaller size of this group.

Notable areas for improvement of the course include spending more time on planning and outlining the goals for specific site visits, increasing the frequency of course meetings above one extended period per week (chosen to facilitate site visits), ensuring students have the opportunity to interact with patients in addition to providers, increasing communication of expectations from the faculty to the students, adding more engineering content to benefit the ID students, ensuring that all students have access to the same suite of software, and an overall desire to push toward the ideation phase of the design process. The open-ended nature of the needs identification process is a departure from theory-based engineering coursework and problem solving. It is likely that select students were longing for more specific deliverables for each site visit whereas some may have been more comfortable with the student-directed discovery process. As the course continues to be offered, there is a desire to increase the cohort size and expand the number of clinical focuses offered to the students. Furthermore, there is a desire to encourage more identified needs to be full-fledged projects following the closing of the course. Many of the needs statements formulated can be altered into a design challenge or sprint prompt. For example, a prompt could be used in sophomore or junior level courses that incorporate design challenges.

## Conclusion

The unique environment and application of this transdisciplinary clinical immersion curriculum offered hands-on experience to students across BME and ID majors. Clinical immersions in the VA environment allowed students to reach out to the community, hear their stories, identify pain points, and finally, propose solutions to solve them. Findings from this course include a promotion for clinical immersion experiences for students, increased visual communication instruction and feedback, and the opportunity for transdisciplinary collaboration between students. These skills are essential for future student success as they begin to enter the workforce. A unique component of this course is the collaboration between the VA, BME and ID, specifically in rural communities. As seen by identified needs progressing to follow-on projects, the results of this ongoing program have made a positive impact on both student career advancement and healthcare advancement.

## Data Availability

Not applicable.

## Code availability

Not applicable.
